# Synthesizing and Evaluating the Photocatalytic and Antibacterial Ability of TiO_2_/SiO_2_ Nanocomposite for Silicate Coating

**DOI:** 10.3389/fchem.2021.738969

**Published:** 2021-09-17

**Authors:** Manh-Cuong Le, Thu-Huong Le, Thanh-Huyen Bui Thi, Quang-Dat Nguyen, Thanh-Ha Do Thi, Minh-Nguyet Tran Thi

**Affiliations:** ^1^ Faculty of Building Material, National University of Civil Engineering, Hanoi, Vietnam; ^2^ Faculty of Chemistry and Environment, Thuyloi University, Hanoi, Vienam

**Keywords:** sol-gel, silicate coating, photocatalytic, antibacterial, nanocomposite

## Abstract

The TiO_2_/SiO_2_ nanocomposite has been synthesized by a sol-gel method and investigated the effect of the SiO_2_ content (0, 5, 10, 15, 20, and 50%) on the rutile-to-anatase phase transition of TiO_2_ NPs. In order to increase the photocatalytic efficiency of the nanocomposite and decrease the price of material, the TiO_2_/SiO_2_ Nc with content SiO_2_ of 15% sample is chosen for preparing silicate coating. The efficiency of photocatalytic MB and antibacterial ability in the air of W silicate coating (adding TiO_2_/SiO_2_ Nc (15%)) achieve almost 100% for 60 h and 94.35% for 3 h, respectively. While the efficiency of photocatalytic MB and antibacterial ability of WO silicate coating (adding commercial TiO_2_/SiO_2_) is about 25–30% for 60 h and 6.02% for 3 h, respectively. The presence of TiO_2_/SiO_2_ Nc (15%) with a larger surface area in W silicate coating can provide increased centers for absorption, photocatalytic reaction, and the contact between sample and bacteria lead to enhance the photocatalytic and antibacterial ability of W silicate coating.

## Introduction

Silicate coating had drawn much attention due to its advances in aging resistance, without or extremely low volatile organic compound emission (Mu et al., 2017; Fagot et al., 2011), less toxic, good resistance to acid and alkali attack (Oleg and Dmitry, 2009), better tolerance to high temperature as well as no combustion (Irfan Khan et al., 2015; Cuong and Thu-Huong, 2021), moisture resistance (Valentina et al., 2018), and anticorrosive coating of concrete or steel structure (Geeta et al., 2001; Parashar et al., 2003). In recent years, research teams have focused on investigating building materials or paints having photocatalytic and antibacterial ability (Pichat, 2013). In addition, there are many studies indicate that components in coating material can impact the photoactivity and bactericidal ability of coating films (Maggos et al., 2007a; Allen et al., 2008; Auvinen and Wirtanen, 2008). In the field of photocatalytic construction and building materials, the titanium dioxide nanoparticle (TiO_2_ NP) is the most widely used photocatalytic bactericidal coating (Allen et al., 2008; Chen and Poon, 2009; Tryba et al., 2015). Chen et al. (2019) have indicated that coating material containing TiO_2_ is a promising self-cleaning building material (Chen and Poon, 2009). Unfortunately, TiO_2_ exhibits limitations such as small surface area, poor absorption property, and facile agglomeration (Jakubickova et al., 2020). In addition, the photo-generated electron and hole of TiO_2_ NPs undergo rapid recombination, which can move to the surface and participate in a redox reaction to generate reactive oxygen species such as hydroxyl radicals (OH∙) and superoxide (O^2-^) (Petronella et al., 2017a; Chen et al., 2019). Particularly, hydroxyl radicals (OH∙) and superoxide (O^2-^) are considered to be dominant species that contribute to the degradation of organic pollution and bacteria (Petronella et al., 2017b). In order to respond to this issue, there are a lot of researchers employ technology for coating or doping TiO_2_ with metallic or non-metallic such as Ag, Cu, Fe, SiO_2_, and ZnO_2_ for preventing rapid recombination of electron and hole, thereby improving the quantum efficiency. Hence, the photocatalytic and antibacterial activity of TiO_2_ can enhance by designing and realizing hybrid nanostructured materials or nanocomposites formed of two or more components (Devi et al., 2020; Ilaria et al., 2020; Luis et al., 2020; Milan et al., 2020). Previous studies on mesoporous silica nanospheres and nanotubes functionalized with titanium dioxide have demonstrated strong enhancement of titanium dioxide photocatalytic performance (Sikora et al., 2017). Moreover, there are also several reported on TiO_2_/SiO_2_ nanocomposite (Nc) material that has shown increases in the specific surface area, porosity, thermal stability, mechanical, antibacterial, and photocatalytic performance (Nilchi et al., 2010; He et al., 2019; Tien et al., 2019; Diana et al., 2020; Pham et al., 2020).

In this paper, we have synthesized TiO_2_/SiO_2_ Nc by a sol-gel method and investigated the effect of the SiO_2_ content (0, 5, 10, 15, 20, and 50%) on the rutile-to-anatase phase transition of TiO_2_ NPs. The WO silicate coating (adding commercial TiO_2_/SiO_2_) and the W silicate coating (adding TiO_2_/SiO_2_ Nc (15%)) thin films have been fabricated by coating on the steel plate (2 × 10 cm) to investigate mechanical properties. The effect of TiO_2_/SiO_2_ Nc on the photocatalytic and antibacterial ability in the air of W silicate coating (adding TiO_2_/SiO_2_ Nc) have determined based on the decomposition of MB under UV-irradiation in a chamber and the detecting and counting the number of colonies on plate count agar (PCA).

## Materials and Methods

### Materials

Liquid glass (mNa_2_O.nSiO_2_. xH_2_O) was purchased from Vietchem Co. Ltd (Vietnam); titanium(IV) isopropoxide (Ti[OCH(CH_3_)_2_]_4_, 97%), tetraethyl orthosilicate (Si(OC_2_H_5_)_4_, 99%), isopropyl alcohol (CH_3_)_2_CHOH, ethanol (C_2_H_5_OH), hydrochloric acid (HCl, 36%), zinc oxide (ZnO, ≥ 99%), calcium carbonate (CaCO_3_, ≥99%), ferrous oxide (Fe_2_O_3_, ≥99%), sodium silicon fluoride (Na_2_SiF_6_, ≥99%), and aluminum oxide (Al_2_O_3_, >98%) were purchased from Xilong Scientific Co., Ltd (China); pigment (white, blue, dark green, chartreuse, black) was purchased from Lanxess AG (Germany); heat-resistant silicon was purchased from Germany; CoapurTM 830W was purchased from Arkema (France); and TiO_2_/SiO_2_ Nc powder was synthesized by our group.

### Synthesis of TiO_2_/SiO_2_ Nanocomposite

**SCHEME 1:**
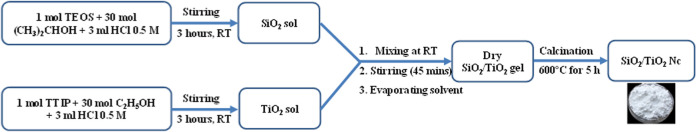
Synthesis of TiO_2_/SiO_2_ Nc (0, 5, 10, 15, 20, and 50%).

The TiO_2_/SiO_2_ nanocomposite (TiO_2_/SiO_2_ Nc) was synthesized (Scheme 1) following a previously reported sol-gel method with the modification (Arun Kumar et al., 2013). SiO_2_ sol was prepared by dropwise 3 ml HCl 0.5 M in the solution of 1 mol TEOS and 30 mol C_2_H_5_OH and stirred for 3 h at room temperature. While TiO_2_ sol was prepared by dropwise 3 ml HCl 0.5 M in the solution of 1 mol TTIP and 30 mol (CH_3_)_2_CHOH and stirred for 3 h at room temperature. The mixed oxide gel (TiO_2_/SiO_2_ gel) was obtained by mixing the SiO_2_ sol with TiO_2_ sol and then stirred for 45 min at room temperature. The solvent was removed by evaporating naturally at room temperature (gel-forming evaporation) until the dry gel was obtained. The dry gel was crushed into a fine powder. Removal of residual organic solvents and stabilization of TiO_2_/SiO_2_ gel were carried out by calcination at 600°C for 5 h. Six nanocomposite sample were synthesized by changing Ti/Si mole percentage including 100:0, 95:5, 90:10, 85:15, 80:20, and 50:50, which shall be referred to as TiO_2_/SiO_2_ Nc (0%), TiO_2_/SiO_2_ Nc (5%), TiO_2_/SiO_2_ Nc (10%), TiO_2_/SiO_2_ Nc (15%), TiO_2_/SiO_2_ Nc (20%), and TiO_2_/SiO_2_ Nc (50%), respectively.

X-ray diffraction spectra were generated to investigate how Ti/Si mole percentage effect the size and composition of the crystal phase of nanometer TiO_2_/SiO_2_ Nc. X-ray diffraction spectra were obtained using D8 Advance (Bruker, Germany) and D5005 (Siemens, Germany). The compositions of the phase were determined through the intensity of the peak as follows (He et al., 2019).

$$W_{A}=\frac{0.886I_{A}}{0.886I_{A}+I_{R}},W_{R}=\frac{I_{R}}{0.886I_{A}+I_{R}}$$ (1)

Where W_A_ and W_R_ represent the mass fraction of anatase and rutile phase, respectively. I_A_ and I_R_ represent the integral intensity of diffraction peaks on the crystal surface of anatase (101) at 2θ = 25.2° and rutile (110) at 2θ = 27.6°, respectively.

The anatase and rutile grain size of TiO_2_/SiO_2_ Nc were calculated using the Debye–Scherrer formula (Diana et al., 2020):

$$d=\frac{0.9\times \lambda }{\beta \times \cos\theta }$$ (2)

Where d is the size of the ordered (crystalline) domains; λ is the X-ray wavelength; β is the line broadening at half the maximum intensity (FWHM) in radians; and θ is the Bragg angle of anatase (101) and rutile (110).

The surface morphology and average particle size of TiO_2_/SiO_2_ Nc (15%) were investigated by transmission electron microscopy (TEM) with a JEOL JEM-2100F. The transmission electron microscopy (TEM) and energy-dispersive X-ray spectroscopy (EDX) were performed with JEOL JEM-2100F.

The chemical structure of TiO_2_/SiO_2_ Nc (15%), TiO_2_ NPs, and SiO_2_ NPs were investigated by FT-IR. FT-IR measurements were taken on a Nicolet 380 spectrometer (Waltham, MA, United States) operated in the mid-IR range of 4,000–400 cm^−1^, with spectra obtained at a spectral resolution of 8 cm^−1^ in transmittance mode. UV-vis absorption spectra were obtained on an UV1800-Japan.

The specific surface area of the TiO_2_/SiO_2_ Nc (15%) and commercial mixture TiO_2_/SiO_2_ (2.55% TiO_2_ and 0.45% SiO_2_) were investigated Quanta chrome Nova Win (United States). Nitrogen (N_2_) adsorption-desorption isotherms were measured at 77K. The Raman spectra were recorded with a LabRAM HR Evolution Spectrometer (Horiba) using a 633 nm laser.

### Preparation of the WO Silicate Coating (Adding Commercial TiO_2_ and SiO_2_) and the W Silicate Coating (Adding TiO_2_/SiO_2_ Nc)

**SCHEME 2:**
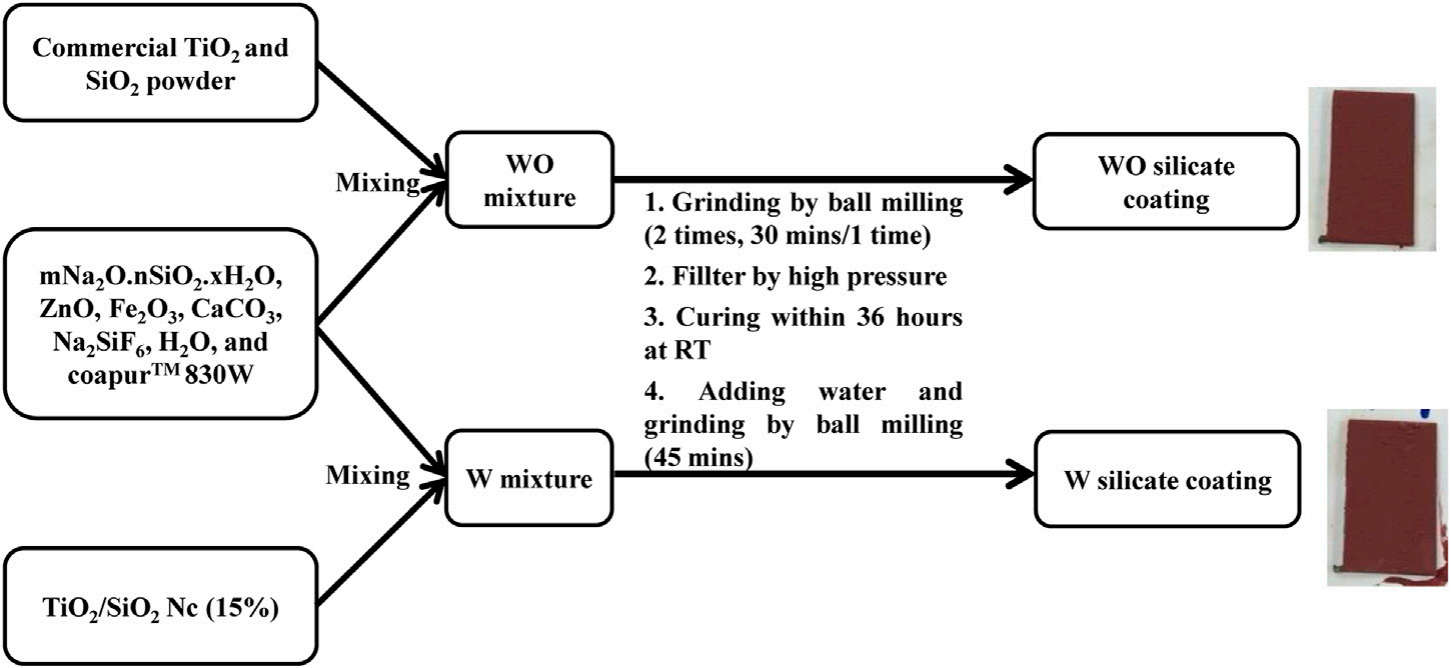
The preparation procedure of WO and W silicate coatings.

WO and W silicate coating were prepared according to the following stages in Scheme 2 and Table 1. The WO silicate coating was prepared by mixing commercial TiO_2_ and SiO_2_ with binder (mNa_2_O.nSiO_2_. xH_2_O) and extender pigments (ZnO, Fe_2_O_3_, CaCO_3_, H_2_O, Na_2_SiF_6_, and CoapurTM 830W). Similarly, W silicate coating was prepared by mixing TiO_2_/SiO_2_ Nc (15%) with binder (mNa_2_O.nSiO_2_. xH_2_O) and extender pigments (ZnO, Fe_2_O_3_, CaCO_3_, H_2_O, Na_2_SiF_6_, and CoapurTM 830W). To create a uniform dispersion for the silicate paint, the ball-milling was used to crush the WO and W silicate coating with a grinding speed is 200–250 rpm for 30 min. The large particles that have not been crushed and marbles were separated by the high-pressure filter method. And then the WO and W silicate coating were cured for 36 h at room temperature to enhance the dehydration and dehydroxylation to form silicate geopolymer (Cuong and Thu-Huong, 2021). The dry WO and W silicate coating were obtained as powder. After that water solvent and WO and W silicate coating powder was mixed and crushed at 150 rpm for 45 min. Finally, the WO and W silicate coating were coated on the steel plate (2 × 10 cm). The WO and W silicate coating thin-film keep overnight to drying and then investigated their mechanical properties.

**TABLE 1:** The formulations of the WO silicate coating and W silicate coating.

WO silicate coating		W Silicate coating	
Ingredient	Amount (%)	Ingredient	Amount (%)
mNa_2_O.nSiO_2_.xH_2_O	28	mNa_2_O.nSiO_2_.xH_2_O	28
ZnO	8	ZnO	8
TiO_2_	2.55	TiO_2_/SiO_2_ Nc (15%)	3
SiO_2_	0.45	Fe_2_O_3_	5
Fe_2_O_3_	5	Na_2_SiF_6_	1
Na_2_SiF_6_	1	CaCO_3_	21
CaCO_3_	21	H_2_O	34
H_2_O	34	Coapur™ 830W	1
Coapur™ 830W	1		

The surface and chemical structures of WO and W silicate coatings were investigated by SEM and FTIR spectra. The viscosity of WO and W silicate coatings solutions was measured by using Brookfield digital viscometer–LVDVE (Brookfield, United States). Drying surface time of WO and W silicate coating thin films were examined according to Vietnam standard of TCVN 2096:1993 Method for determination of dry state and dry time. The thicknesses of WO and W silicate thin films were measure by using Coating thickness gause PCE-CT 28 (PCE group, England). The bending strength of WO and W silicate coating thin films was examined by using TQC Sheen SP 1820 (TQC Sheen, England) according to an ASTM D522 cylindrical mandrel bend test. Film hardness by Pencil of WO and W silicate coating thin films were examined by using TQC Sheen VF 2377 (TQC Sheen, England) according to an ASTM D3363 film hardness by Pencil test. Impact resistance of WO and W silicate coating thin films were examined by using Laryee FIT1130 (Laryee Technology, China) according to Vietnam standard of TCVN 21000–1:2007 paint and varnishes–Rapid–deformation (impact resistance) test.

### Evaluating the Photocatalytic Ability of WO and W Silicate Coating

**FIGURE 1:**
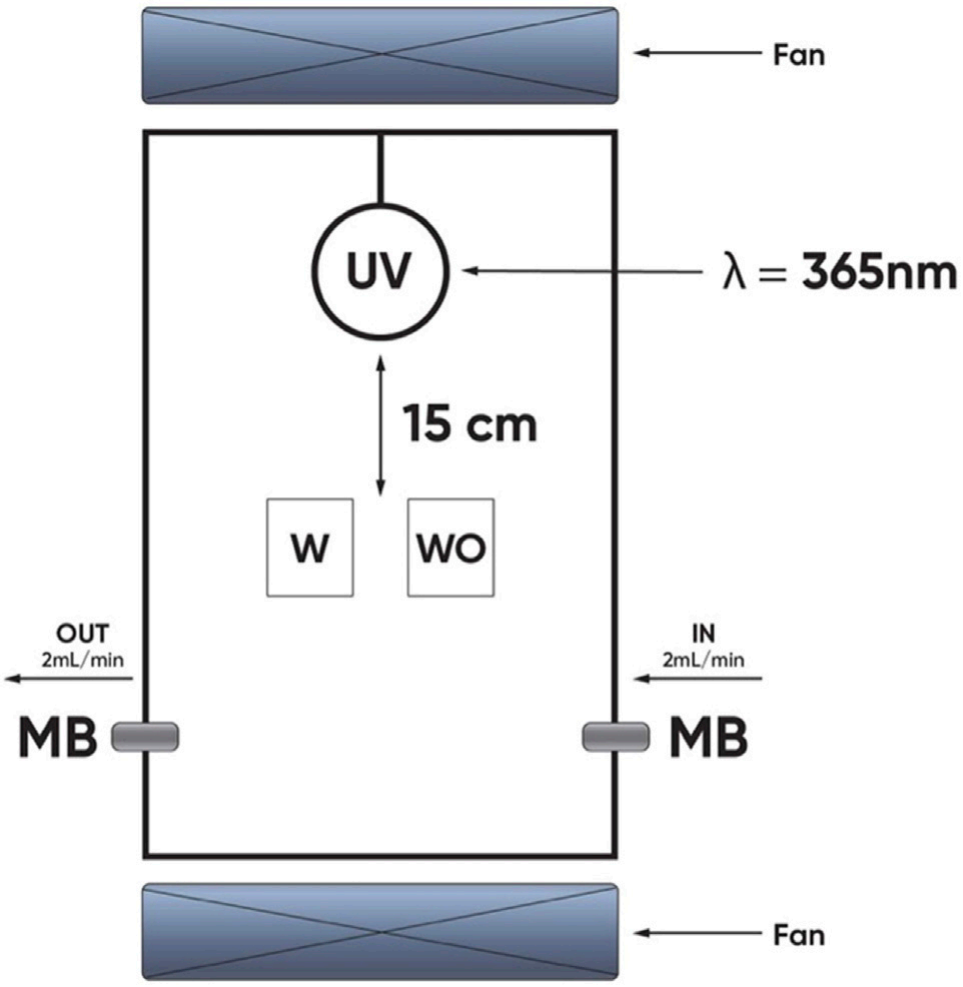
Schematic diagram of the photocatalytic experimental of the WO and W silicate coating.

Photocatalytic test of WO and W silicate coating was performed for decomposition of methylene blue (MB) under UV-irradiation in the chamber (Figure 1). This chamber was equipped with a UV lamp (365 nm). The irradiation intensity is 1 mW/cm^2^. The WO (adding commercial TiO_2_/SiO_2_) and W (adding TiO_2_/SiO_2_ Nc**)** silicate coatings were coated on the plastic beaker (the area surface was approximately 16 cm^2^) and left for drying overnight. MB 5 ppm solutions were poured into the plastic tray with WO and W silicate coating on the surface. Then, the plastic beaker was poked into a small hole and sealed by a cotton ball to let the MB flow through with a flow rate of 2 ml/min (Asahi et al., 2001; Ichimura et al., 2005). The system was placed under UV- irradiation in the chamber (365 nm) with an irradiation intensity measured of 1 mW/cm^2^ for 60 h (Deibold, 2003; Maggos et al., 2007b). By measuring the concentration of MB before and after flowing through plastic beaker, we can evaluate the efficiency of the photocatalytic of the WO and W silicate coating. UV-DR3900 spectrometer (Hach Co., Ltd., United States) was used to obtain UV-vis absorption spectrum of MB at 660 nm (Axel and Jan 1998; Tachikawa et al., 2007).

### Evaluating the Antibacterial in Air Ability of the WO and W Silicate Coating

**FIGURE 2:**
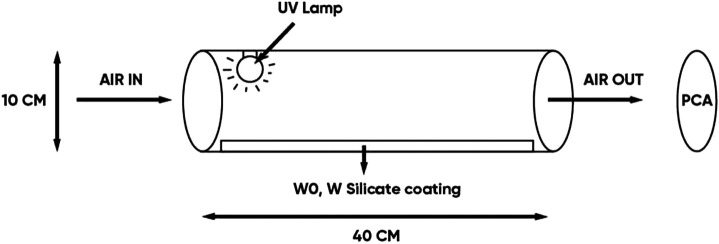
Schematic diagram of the antibacterial experimental of the WO and W silicate coating.

The antibacterial in air efficiency of the WO and W silicate coating material (Figure 2) was determined based on the method of detecting and counting the number of colonies on plate count agar (PCA). The equipment system includes a quartz tube (diameter 10 cm, length 40 cm) coated with WO and W silicate coating on its surface, UV lamp (power, 45W) placed in a quartz tube, Spin Air device (IUL), and PCA (5 g/L casein peptone, 2.5 g/L yeast extract, 1 g/L glucose, 9–18 g/L Agar, pH = 7). The air passed through quartz tube under UV–irradiation (nm) and in contact with WO and W silicate coating on surface quartz tube from 1 to 3 h. The air in the quartz tube was sucked out by the vacuum of the Spin Air device. The out-put air hit the surface of the PCA. PCA was cultured at 37°C for 24 ± 2 h. After that, we counted the total number of colonies growing on the PCA plate. The antibacterial efficiency was determined by comparing the results of the total number of colonies growing on the PCA of the air samples passing through a quartz tube (coated with WO and W silicate coating) with the air samples passing through the quartz tube (without coating material) (Allen et al., 2008).

## Results and Discussion

### Characterization of TiO_2_/SiO_2_ Nc

**FIGURE 3:**
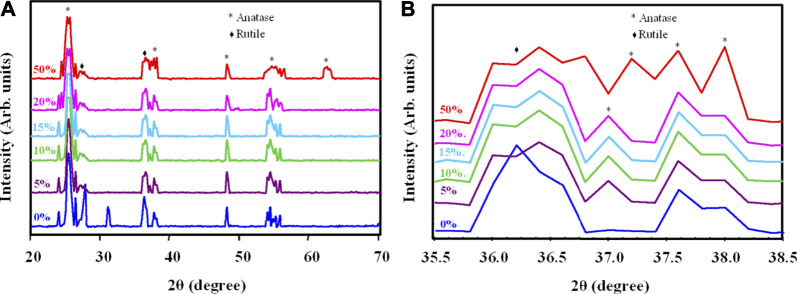
**(A)** X-ray diffraction of TiO_2_/SiO_2_ Nc (0–50%) sintered at 600°C and **(B)** Change in the peaks at 36.2° and 37.6° in X-ray diffraction, indicating the reduce crystallite size.

Figures 3A,B shows the XRD results of the TiO_2_/SiO_2_ Nc (0–50%) samples heated at 600 °C for 5 h. The XRD of TiO_2_/SiO_2_ Nc (0%) sample has peaks at 2θ of 25.2, 27.6, 36.2, 37.6, 48.0, 53.5, 55.0 and 62.6° correspond to the TiO_2_ with anatase (JCPDS 84–1,286) and rutile phases ((JCPDS 76–0,649) (Arun Kumar et al., 2013; Billy et al., 2019). However, the presence of SiO_2_ is not indicated from patterns of TiO_2_/SiO_2_ Nc (5–50%) samples probably due to the high crystalline TiO_2_ from to cover amorphous SiO_2_, which presents a broad peak at 20–30° (Arun Kumar et al., 2013). Moreover, as the SiO_2_ content increases from 5 to 50%, the rutile peak at 36.2° and anatase peak at 37.6° became broader (Figure 3B) (Billy et al., 2019). There is three cause of line broadening of XRD reflections: crystallite size, lattice strain, and lattice mistake (Okada et al., 2001). However, the broadening of XRD reflections is believed to be due mainly to the small crystallite size, and the other possible causes are not addressed. The crystal size and composition of the crystal phase of TiO_2_/SiO_2_ Nc (0–50%) samples are determined through the integral intensity and the line broadening at half the maximum intensity of diffraction peaks anatase (101) at 2θ = 25.2° and rutile (110) at 2θ = 27.6° (Okada et al., 2001)

As shown in Table 2, the mass fraction of anatase increases from 64.4 to 90.7% when the SiO_2_ content increases from 0 to 50%. The anatase grain size decreases from 20.0 to 18.2 nm when the SiO_2_ content increases from 0 to 50%. The rutile grain size also decreases from 20.0 to 16.8 nm when the SiO_2_ content increases from 0 to 50%. Alumina, silica, and zirconia have been used to stabilize anatase (Hanaor and Sorrell, 2011). Akhtar et al. have reported that the addition of SiO_2_ drastically altered the morphology of TiO_2_ particles from polyhedral to spheroidal, increased the extent of aggregation, increased the specific surface area, reduced the primary particle size, and decreased the rutile content (Akhtar et al., 1992). In particular, Okada et al. (2001) have concluded that the formation of this amorphous SiO_2_ surface layer was considered to be important in retarding the anatase to rutile phase transition by suppressing diffusion between anatase particles in direct contact and limiting their ability to act as surface nucleation sites for rutile. Thus, we suggest that the increase of SiO_2_ content in TiO_2_/SiO_2_ Nc prevents the crystalline transition to the rutile phase, results in the content of the anatase phase increased gradually from 64.4 to 90.7%. Besides, the adding of SiO_2_ into TiO_2_ could retard the growth of nanoparticles and reduce the anatase grain size from 20.0 to 18.2 nm. The SEM images (Supplementary Figure S1) show the effect of SiO_2_ content on the reduced particle size of TiO_2_/SiO_2_ Nc (0–50%). These discoveries have great significance in the synthesis of nanomaterial used for photocatalytic processes. In this research, TiO_2_/SiO_2_ Nc (20% or 50%) samples show the composition of the anatase phase of 89.3% or 90.7%, respectively, which not much larger than TiO_2_/SiO_2_ Nc (15%) sample (88.2%). Although TiO_2_/SiO_2_ Nc (20% or 50%) samples contain the high composition of the anatase phase, the amount of TiO_2_ is only 80% or 50%, which leads to a decrease in the photocatalytic efficiency of the nanocomposite. Thus, the TiO_2_/SiO_2_ Nc (15%) sample which contains SiO_2_ of 15% and TiO_2_ of 85% is chosen for preparing silicate coating.

**TABLE 2:** The composition of the anatase, rutile phase, and the crystallite size of the TiO_2_/SiO_2_ Nc (0–50%).

Percent of SiO_2_ (%)	Anatase		Rutile	
	Mass fraction of anatase (%)	Anatase grain size (nm)	Mass fraction of rutile (%)	Rutile grain size (nm)
0	64.4	20.0	35.6	20.8
5	76.8	20.2	23.2	20.0
10	83.9	20.1	16.1	19.8
15	88.2	20.1	11.8	19.4
20	89.3	19.5	10.7	19.3
50	90.7	18.2	9.3	16.8

**FIGURE 4:**
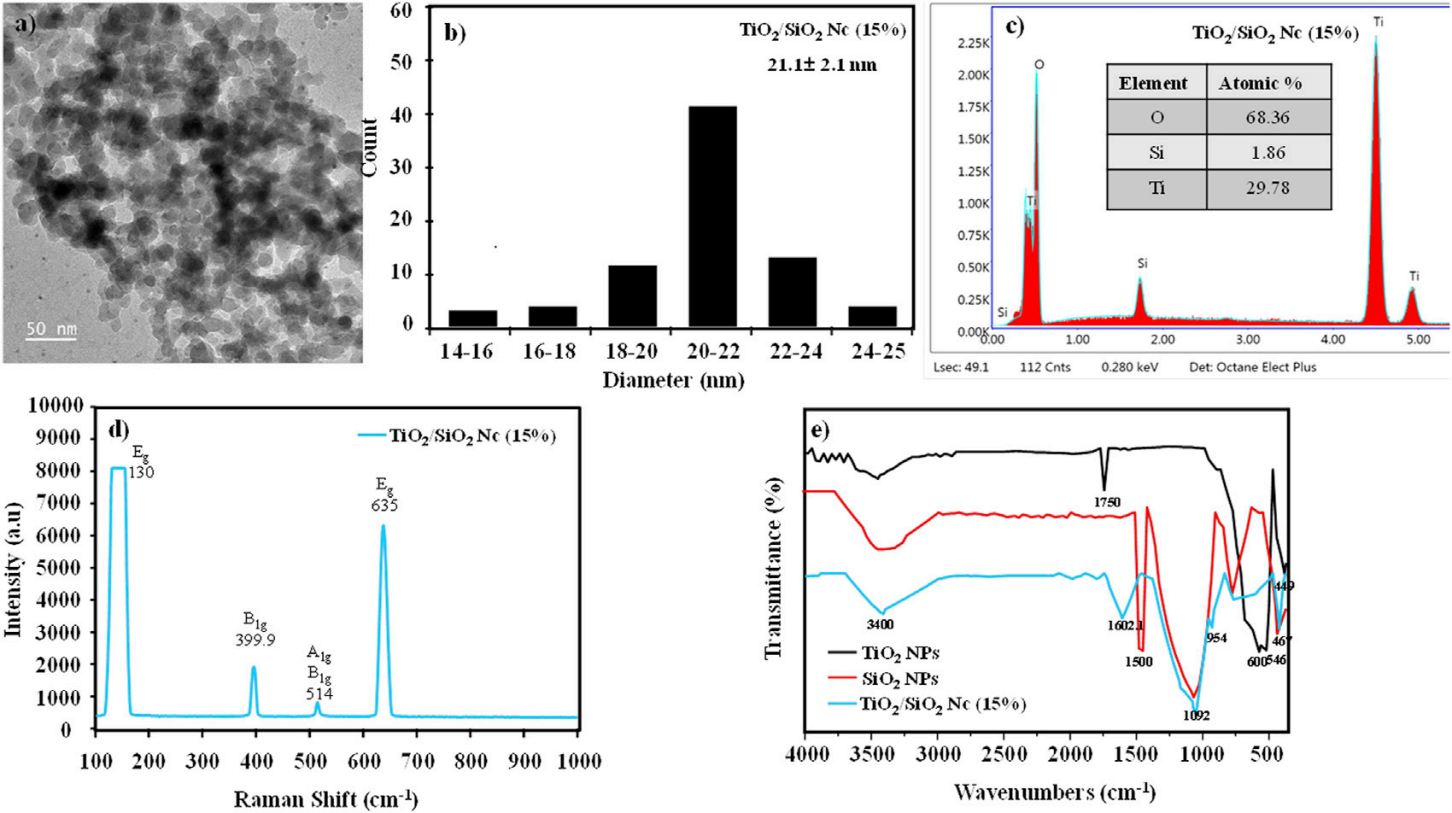
**(A,B)** Transmission electron microscopy (TEM) and size distribution of TiO_2_/SiO_2_ Nc (15%); **(C)** Energy dispersive X-Ray (EDX) spectra of TiO_2_/SiO_2_ Nc (15%); **(D)** Raman spectra of TiO_2_/SiO_2_ Nc (15%); **(E)** FT-IR spectra of TiO_2_/SiO_2_ Nc (15%) in comparison with the TiO_2_ NPs, and SiO_2_ NPs.

The surface morphology and particle size of TiO_2_/SiO_2_ Nc (15%) are evaluated by TEM (Figures 4A,B). The TEM image clearly shows that the spherical particle of the TiO_2_/SiO_2_ Nc (15%) sample is formed with the average size of 21.1 ± 2.1 nm. The compositions of the TiO_2_/SiO_2_ Nc (15%) sample (Figure 4C) are analyzed by energy-dispersive X-ray spectroscopy (EDX). EDX result shows that TiO_2_/SiO_2_ Nc (15%) sample composes of Ti element (29.78% from TiO_2_/SiO_2_ Nc), Si element (1.86% from TiO_2_/SiO_2_ Nc), and O element (68.36% from TiO_2_/SiO_2_ Nc). The Si element is detected in the EDX result which shows that SiO_2_ has existed in the nanocomposite.

The Raman spectra of TiO_2_/SiO_2_ Nc (15%) are shown in Figure 4D. The peaks observed at 130, 635, 399.9, and 514 cm^−1^ are attributed to vibrational modes E_g_, B_1g_, and A_1g_, respectively, characteristic of the anatase phase (Billy et al., 2019). Moreover, these peaks show narrow, which indicating a better crystallization of anatase in the nanocomposite.

The chemical structure of TiO_2_/SiO_2_ Nc (15%), TiO_2_ NPs, and SiO_2_ NPs were investigated by FTIR spectra (Figure 4E). The FTIR spectra three samples show a wide absorption spectrum at 3,000–3,800 cm^−1^, which is the O–H bond and relates to re-adsorb water from the ambient atmosphere on the surface during sample preparation for FT-IR analysis (Pal et al., 2014; Wojciechowki et al., 2015). Spectral lines at 1,500 (red line) and 1,602.1 cm^−1^ (green line) are oscillations of H–O–H bonds and are believed to adsorb water on Si–O or Ti-O bonds. The peaks at 600 and 546 cm–1 (black line) and 449 cm^−1^ (blue line) are caused by the oscillation of Ti–O–Ti and Si–O–Si bonds (Di Crescenzo et al., 2013; Pal et al., 2014). The weak peak at 954 cm-1 reveals the interaction between titania and silica to form Ti-O-Si bonds in nanocomposite (Lee et al., 2007; Rees et al., 2007; Cuong and Thu-Huong, 2021). The formation of the Ti-O-Si bond confirms the presence of SiO_2_ around TiO_2_, which would prevent the growth of TiO_2_ particles, or reduce crystal size and particle size of TiO_2_/SiO_2_ Nc as obtained in XRD and SEM results.

**FIGURE 5:**
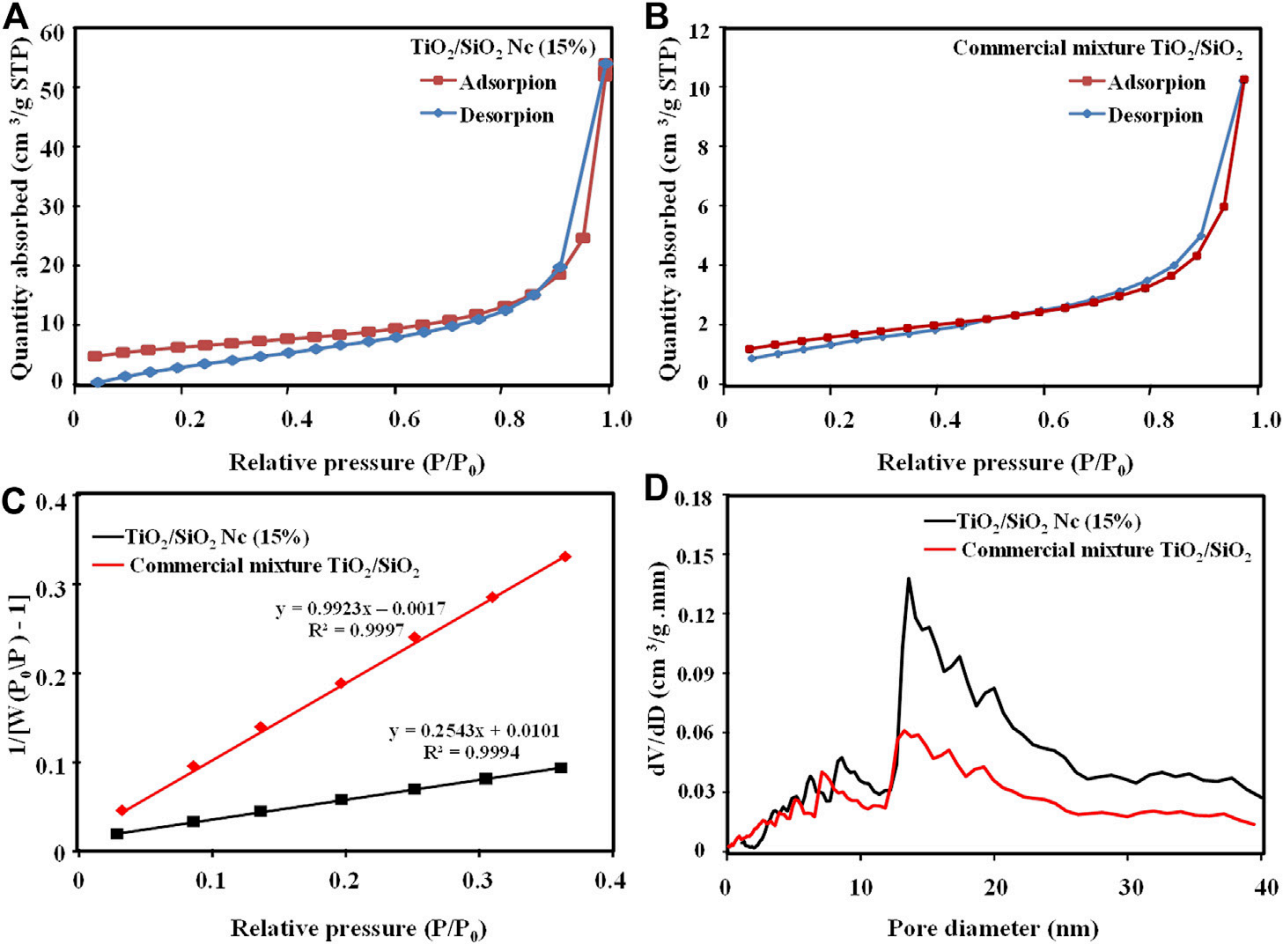
Nitrogen adsorption isotherm pattern of TiO_2_/SiO_2_ Nc (15%) **(A)** and commercial mixture TiO_2_/SiO_2_ **(B)** *,* BET plot of TiO_2_/SiO_2_ Nc (15%) and commercial mixture TiO_2_/SiO_2_ **(C)**, and BJH pore size distribution **(D)**.

The nitrogen adsorption-desorption isotherms and BJH (Barrett–Joyner–Halenda) pore size distribution of the TiO_2_/SiO_2_ Nc (15%) and commercial mixture TiO_2_/SiO_2_ are shown in Figure 3. In Figures 5A,B, the adsorption-desorption isotherms of TiO_2_/SiO_2_ Nc (15%) and commercial mixture TiO_2_/SiO_2_ are IUPAC type IV, which indicates the presence of mesoporous material with hysteresis in high relative pressure (Aguado et al., 2006). Figure 5D shows the pore size distribution of TiO_2_/SiO_2_ Nc (15%) and commercial mixture TiO_2_/SiO_2_ smaller than 20 nm. Hence, the BJH analysis demonstrates that the TiO_2_/SiO_2_ Nc (15%) and commercial mixture TiO_2_/SiO_2_ exhibit disordered mesoporous structures. The surface area of TiO_2_/SiO_2_ Nc (15%) is 132.9 m^2^/g (Figure 5C and Table 3), which is most 4 times larger than that of commercial mixture TiO_2_/SiO_2_ (31.4 m^2^/g). The increased specific surface area mainly results from the large specific surface area of SiO_2_ and nano size of TiO_2_/SiO_2_ Nc (15%). The TiO_2_/SiO_2_ Nc (15%) with a larger surface area can provide increased centers for absorption and photocatalytic reaction. In addition, high BET surface area is also clearly beneficial for contact between sample and bacteria lead to enhance the antibacterial ability.

**TABLE 3:** The surface area and BJH adsorption pore size of TiO_2_/SiO_2_ Nc (15%) and commercial mixture TiO_2_/SiO_2_.

Sample	Surface area (m^2^/g)	BJH pore size (nm)
TiO_2_/SiO_2_ Nc (15%)	132.9	6.0, 8.0, and 13.2
Commercial mixture TiO_2_/SiO_2_	31.4	5.8, 7.7 and 12.0

### Characterization of WO and W Silicate Coating

**FIGURE 6:**
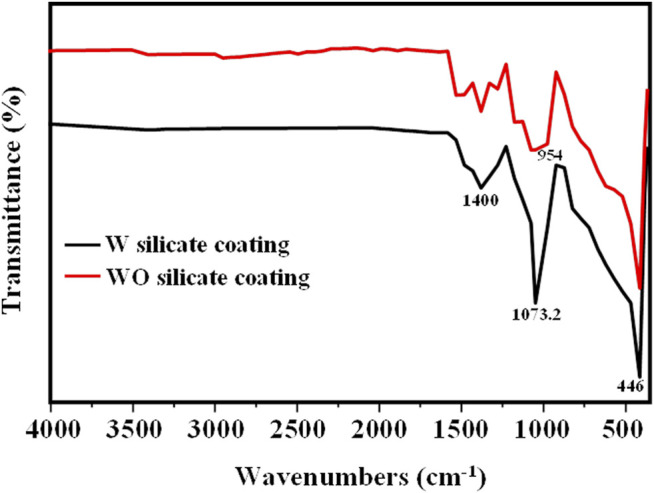
Infrared absorption spectroscopy (FT-IR) of WO and W silicate coating samples.

The chemical structure of WO and W silicate coatings has been investigated by FT-IR spectroscopy as shown in Figure 6. In both WO and W silicate coatings, a strong and relatively broad peak centered at 1,073.2 cm^−1^ is observed. This band can be assigned asymmetrical elongation of Si–O–Si superimposed with Si–O–Na band oscillations resulting from the dry component interacting with the sodium silicate binder (OssWald and Fehr, 2006; Cuong and Thu-Huong, 2021). In both paints, some commonly observed performance traits can be identified as calcium carbonate (CaCO_3_), which shows strong absorption around 1,400–1,427 cm^−1^ (asymmetrical elongation of CO_3_^2-^) (Cuong and Thu-Huong, 2021). Another common feature is the presence of a wide absorption band at 446 cm^−1^, arising from the Ti–O–Ti extended oscillation of TiO_2_ pigments (Di Crescenzo et al., 2013; Pal et al., 2014). In addition, the FT-IR result of W silicate coating shows the shoulder peak at 954 cm^−1^ that reveals the interaction between titania and silica to form Ti-O-Si bonds in nanocomposite (Lee et al., 2007; Rees et al., 2007; Cuong and Thu-Huong, 2021).

**FIGURE 7:**
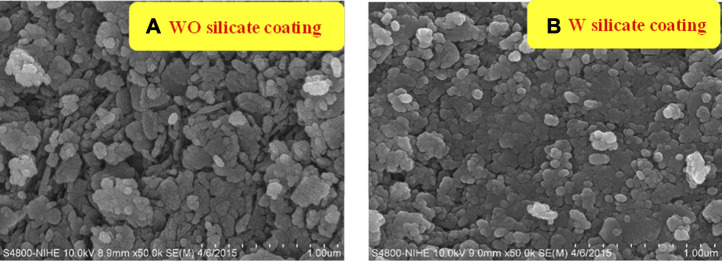
SEM image of WO **(A)** and W **(B)** silicate coatings.

The effect of TiO_2_/SiO_2_ Nc (15%) on the surface morphology of W silicate coating is investigated by measuring the SEM of WO silicate coating (with commercial TiO_2_ and SiO_2_ powder) and W silicate coating (with TiO_2_/SiO_2_ Nc (15%) (Figures 7A,B). In Figure 7A, the SEM results of the WO silicate coating show the formation of agglomerates of larger particles, while SEM of W silicate coating (Figure 7B) clearly shows isolate nanoparticles with an average size of 100 nm. According to the nitrogen adsorption-desorption isotherms (Figures 5A,B; Table 3), the TiO_2_/SiO_2_ Nc (15%) with a mesoporous structure and large surface area (132.9 m^2^/g) makes their ability to well-dispersed and interact with binder (liquid glass**)** and extender pigments, which lead to the formation of isolate nanoparticles of W silicate coating This is an important feature indicating that W silicate coating with adding TiO_2_/SiO_2_ Nc (15%) has the potential to be photocatalytic and bactericidal materials.

### Mechanical Properties of WO and W Silicate Coating

The mechanical properties results of WO and W silicate coating are shown in Table 4.

**TABLE 4:** The mechanical properties of WO and W silicate coating.

	WO silicate coating	W silicate coating
Viscosity (cP)	900	924
Drying surface time, min	12	10
Thickness, μm	60	59
Impact resistance, J	30	30
Bend strengh, mm	3	3
Film hardness by pencil, H	7	7

As shown in Table 4, the viscosity of WO silicate coating (900 cP) is smaller than W silicate coating due to the presence of TiO_2_/SiO_2_ Nc (15%) with a large BET surface area of 132.9 m^2^/g. These results lead to drying surface time and the thickness results of WO silicate thin film are higher than W silicate thin film. However, the impact resistance, bend strength, and film hardness by pencil of WO and W silicate coating are similar. Thus, the above results show that the addition of TiO_2_/SiO_2_ Nc (15%) does not change the mechanical properties compared with WO silicate coating (adding commercial TiO_2_ and SiO_2_).

### Evaluating the Photocatalytic Ability of WO and W Silicate Coating

The decomposition percentage MB of the WO and W silicate coating is calculated by following Eq. 3:

$$\%=\frac{C_{input}-C_{output}}{C_{input}}$$ (3)

**FIGURE 8:**
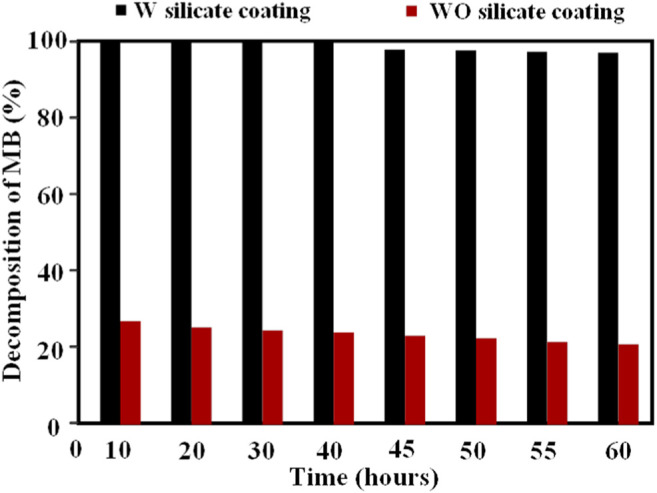
Decomposition percentage MB of the WO and W silicate coatings.

In which, C_input_ is the input concentration of MB (ppm) and C_output_ is the output concentration of MB (ppm). The results of the photocatalytic performance of the WO and W silicate coatings are given in Figure 8; Table 5.

**TABLE 5:** Results of the photocatalytic performance of the WO and W silicate coatings.

Time (h)	Input concentration of MB (ppm)	Output concentration of MB (ppm)	
		WO silicate coating	W Silicate coating
10	5	3.650	−
20	5	3.735	−
30	5	3.770	−
40	5	3.800	−
45	5	3.840	0.120
50	5	3.875	0.130
55	5	3.920	0.145
60	5	3.955	0.160

**SCHEME 3:**
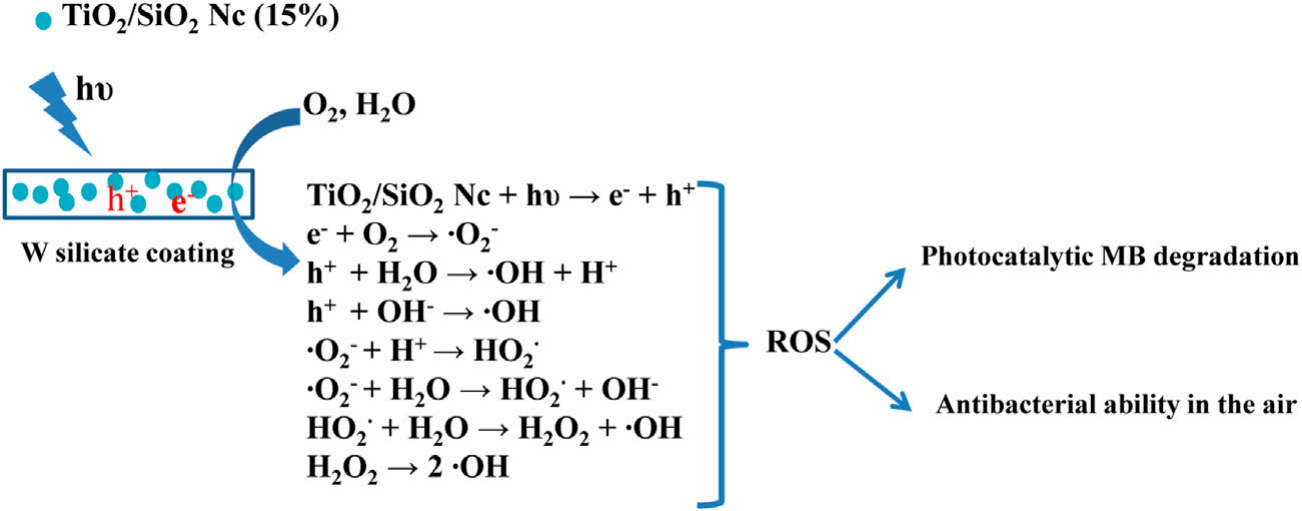
Photocatalytic methylene blue degradation and antibacterial in the air of W silicate coating.

Table 5 and Figure 8 show that the efficiency of the photocatalytic MB of the W silicate coating achieves almost 100% for 40 h, after 20 h the efficiency of the photocatalytic MB decrease to 96.0%. While the efficiency results of the WO silicate coating sample (adding commercial TiO_2_ and SiO_2_) are just about 25–30%. The decreased in decomposition percentage of WO silicate coating compared with the result of W silicate coating is likely to be attributed to the presence of TiO_2_/SiO_2_ Nc (15%). As Allen et al. (2008) have reported that the photocatalytic behavior of TiO_2_ NPs due to converting photons into excitons under illumination, which participate in redox reactions and generate reactive oxygen species (ROS), such as hydroxyl radicals (OH·), oxide anion radicals (∙O_2_^−^), and hydrogen peroxide (H_2_O_2_) (Ilaria et al., 2020). These ROS are considered to be dominant species that contribute to the degradation of organic pollution and bacteria. However, the photogenerated electrons and holes undergo rapid recombination, which is not conductive to the production of ROS. To overcome these issues, SiO_2_ with a larger surface area, high thermal stability, multi-channel structure, and stable chemical properties (Thu et al., 2019) are used to coat TiO_2_ for trapping the photogenerated electrons, which can prevent the rapid recombination of electrons and holes. In addition, the performance photocatalytic of TiO_2_ strongly depends on the mass fraction of the anatase phase (Arun Kumar et al., 2013). The presence of SiO_2_ in TiO_2_/SiO_2_ Nc (15%) not only inhibits the anatase to rutile phase transformation but also enhances the photocatalytic performance of TiO_2_ NPs (Cendrowski et al., 2013). Moreover, the surface area of TiO_2_/SiO_2_ Nc (15%) is 132.9 m^2^/g (Table 3), which is almost 4 times larger than that of commercial mixture TiO_2_/SiO_2_ (31.4 m^2^/g) (Scheme 3). The TiO_2_/SiO_2_ Nc (15%) with a larger surface area can provide more centers for absorption and photocatalytic reaction than commercial mixture TiO_2_/SiO_2_. Therefore, we can assert that the photocatalytic MB behavior and performance of W silicate coating with adding TiO_2_/SiO_2_ Nc (15%) are higher than WO silicate coating with adding commercial mixture TiO_2_/SiO_2_. Although the durability of the photocatalytic activity of silicate coating has been tested for 60 h, the potential of W silicate coating materials is huge and promising due to the high decomposition percentage MB (96–100%).

### Evaluating the Antibacterial Ability in the Air of WO and W Silicate Paints

The antibacterial efficiency in the air of WO and W silicate coatings was determined by comparing the results of the number of colonies growing on the PCA of the air passing through a quartz tube coating WO and W silicate coating from 1–3 h with the results of the number of colonies growing on the PCA of the air passing through a quartz tube without coating material (Table 6).

**TABLE 6:** Results of the antibacterial efficiency in air of the WO and W silicate coatings.

Time, h	WO silicate coating, %	W Silicate coating, %
0	0	0
1	5.74	87.61
2	5.97	94.35
3	6.02	94.35

**FIGURE 9:**
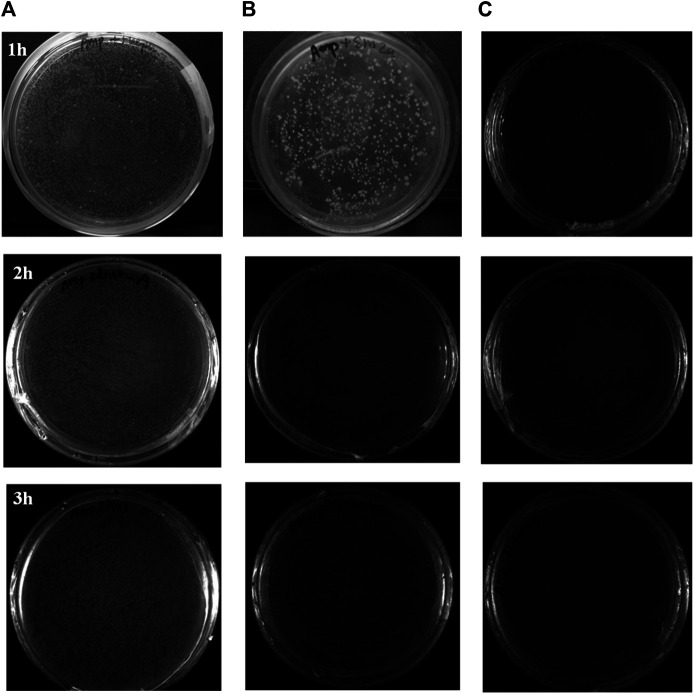
The antibacterial evaluation on plate count agar (PCA) of the air passing through a quartz tube without coating material **(A)**, WO silicate coating **(B)**, and W silicate coating **(C)** from 1 to 3 hours.

The antibacterial ability in the air of W silicate coating from 1–3 h (Figure 9; Table 6) shows that the first the antibacterial efficiency increases rapidly and reached a quite high efficiency of 87.61% for 1 h and then increases slowly after 2–3 h (94.35%). While WO silicate coating shows the opposite trend, the antibacterial efficiency does not change much within 3 h (5.74–6.02%). Under UV lamp (365 nm), the TiO_2_/SiO_2_ Nc (15%) in W silicate coating generate reactive oxygen species (ROS) such as hydroxyl radicals (OH^•^), oxide anion radicals (∙O^2−^), and hydrogen peroxide (H_2_O_2_) as shown in Scheme 3 (Cendrowski et al., 2013; Chen et al., 2019; Thu et al., 2019). Moreover, the presence of SiO_2_ in TiO_2_/SiO_2_ Nc (15%) enhances the absorption properties, that is, a larger amount of bacteria in air can adsorb on W silicate coating. Thus, the TiO_2_/SiO_2_ Nc (15%) with a larger surface area in W silicate coating is beneficial for the contact between sample and bacteria lead to enhance the antibacterial in air ability compared with WO silicate coating.

## Conclusion

In conclusion, TiO_2_/SiO_2_ Nc with different SiO_2_ contents (0, 5, 10, 15, 20, and 50%) were prepared by sol-gel method. The addition of SiO_2_ inhibited the phase transition of TiO_2_ nanoparticles from anatase to rutile when sintering at the same temperatures of 600°C. The TiO_2_/SiO_2_ Nc (15%) sample with anatase content accounting for 88.2% is chosen for preparing silicate coating (WO and W silicate coating). The photocatalytic potential and antibacterial ability in the air of WO and W silicate coatings were investigated based on the decomposition of MB under UV-irradiation in a chamber and the method of detecting and counting the number of colonies on plate count agar (PCA). The efficiency of the photocatalytic MB of the W silicate coating achieves almost 100% for 40 h, after 20 h the efficiency of the photocatalytic MB decrease to 96.0%. While the efficiency results of the WO silicate coating sample (adding commercial TiO_2_ and SiO_2_) are just about 25–30%. The antibacterial ability in the air of W silicate coating shows that the first the antibacterial efficiency increases rapidly and reached a quite high efficiency of 87.61% for 1 h and then increases slowly after 2–3 h (94.35%). While WO silicate coating shows the opposite trend, the antibacterial efficiency does not change much within 3 h (5.74–6.02%). Therefore, we can assert that the TiO_2_/SiO_2_ Nc (15%) with a larger surface area in W silicate coating is beneficial for the contact between sample and bacteria lead to enhance the photocatalytic activity and antibacterial in air ability compared with WO silicate coating. W Silicate coating [adding TiO_2_/SiO_2_ Nc (15%)] with self-cleaning active UV has been developed. Moreover, it is superior to organic paints due to its high heat resistance, long life, and low price, which has potential application in the environment-friendly paint industry.

## Data Availability

The original contributions presented in the study are included in the article/Supplementary Material, further inquiries can be directed to the corresponding authors.
