# Supplementary figures and images for: Synthesizing and Evaluating the Photocatalytic and Antibacterial Ability of TiO2/SiO2 Nanocomposite for Silicate Coating
Source: Front Chem. 2021 Sep 17;9:738969. doi: 10.3389/fchem.2021.738969 (PMC8485069; doi:10.3389/fchem.2021.738969)

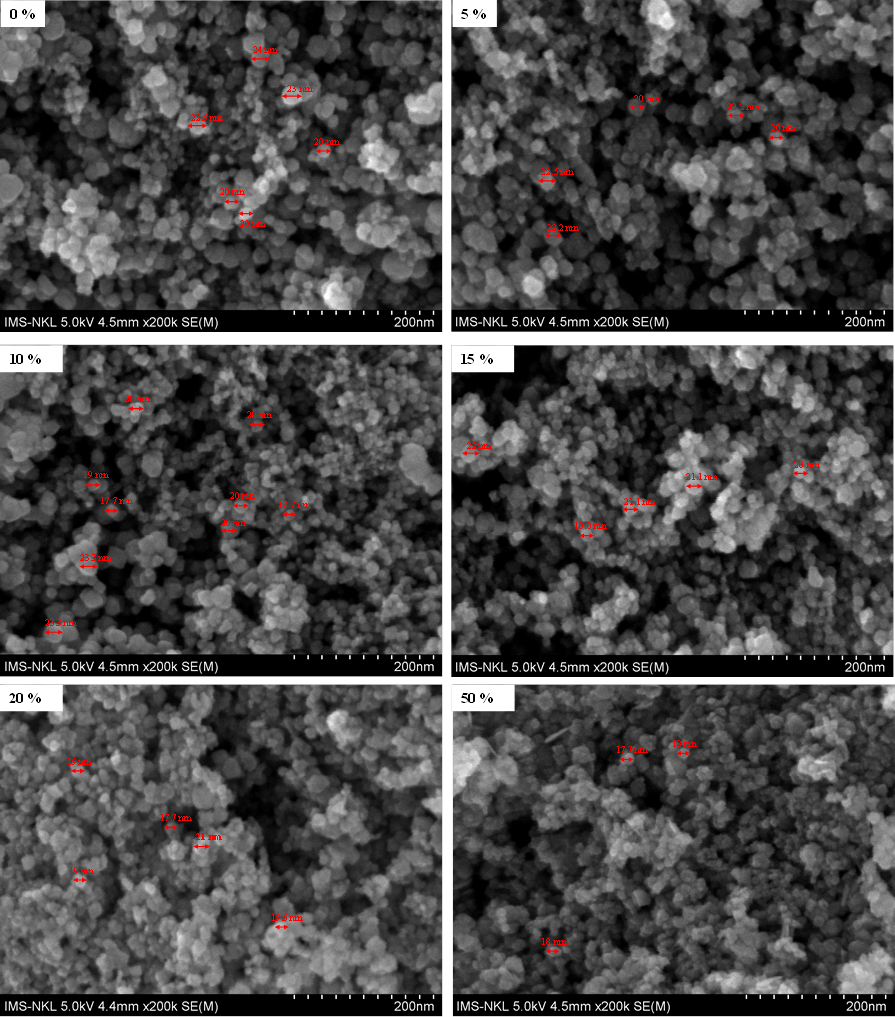

Supplement: Supplementary file 1 [file Image1.JPEG]
