# Supplementary material for: Synthesizing and Evaluating the Photocatalytic and Antibacterial Ability of TiO2/SiO2 Nanocomposite for Silicate Coating
Source: Front Chem. 2021 Sep 17;9:738969. doi: 10.3389/fchem.2021.738969 (PMC8485069; doi:10.3389/fchem.2021.738969)
Supplement: Supplementary file 2 [file DataSheet1.pdf]

Synthesizing and Evaluating the Photocatalytic and Antibacterial Ability of TiO_2/SiO_2 Nanocomposite for Silicate Coating

Manh-Cuong Le [1]*, Thu-Huong Le [2]*, Thanh-Huyen Bui Thi[1], Quang-Dat Nguyen[1], Thanh-Ha Do Thi[1], Minh-Nguyet Tran Thi[1]

[1]Faculty of Building Material, National University of Civil Engineering, Ha Noi/100000, Viet Nam.

[2]Faculty of Chemistry and Environment, Thuyloi University, Ha Noi/100000, Viet Nam.

*** Correspondence:** Manh-Cuong Le and Thu-Huong Le
email: cuonglm@nuce.edu.vn

lethuhuong@tlu.edu.vn

1. Results and Discussion

The surface morphology and particle size of TiO_2/SiO_2 Nc (0–50%) were evaluated by SEM (Figure S1). The SEM image clearly shows that the isolated spherical particle of the TiO_2/SiO_2 Nc (0-50%) samples are formed. Second, when the SiO_2 content in the nanocomposite sample increases from 0 to 15%, the SEM results of four samples TiO_2/SiO_2 Nc (0-15%) show a the particle size samples decreases slowly. In contrast, the SEM results of two samples TiO_2/SiO_2 Nc (20-50%) show decreases rapidly. These SEM results are consistent with the XRD results reported above. As the SiO_2 content increases from 0 to 50%, the anatase and rutile grain size decrease. Thus, we suggest that the increased of SiO_2 content in TiO_2/SiO_2 Nc prevent the crystalline transition to the rutile phase, results in retard the growth of nanoparticles and reduce the anatase grain size from 20.0 to 18.2 nm and rutile grain size from 20.8 to 16.8 nm.

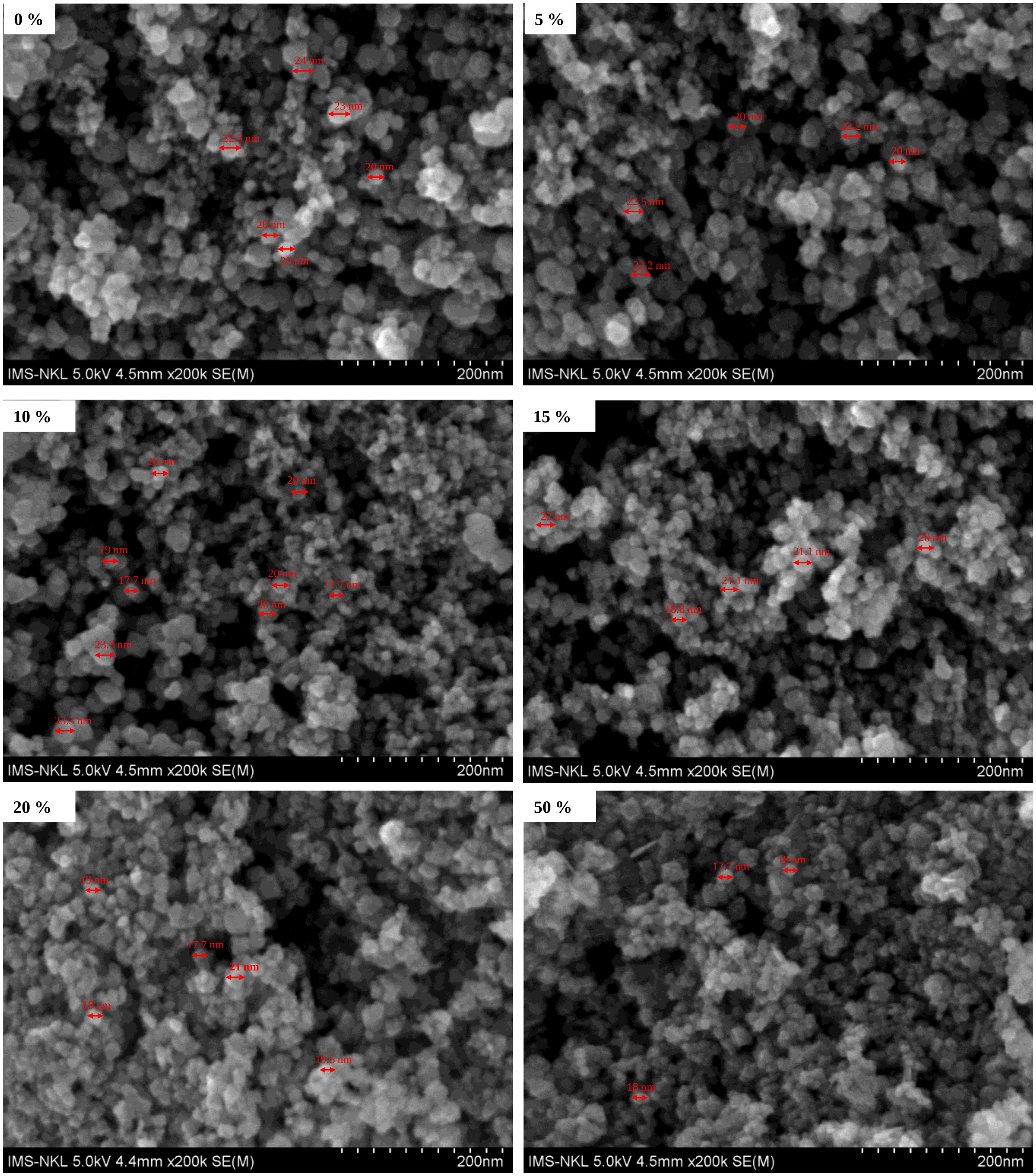

Figure S1. SEM images of TiO_2/SiO_2 Nc (0-50%) samples.

This is a provisional file, not the final typeset article
